# Impact of Shooting Distance on Oculomotor Strategy in Basketball Shooting

**DOI:** 10.1002/ejsc.70156

**Published:** 2026-03-12

**Authors:** Alessandro Piras

**Affiliations:** ^1^ Department for Life Quality Studies University of Bologna Bologna Italy

## Abstract

The purpose of this study was to examine the role of eye movements during basketball shooting from various distances. Fourteen male basketball players, equipped with an eye tracker and an inertial sensor, performed 40 shots on a basketball court, divided into free throws and 3‐point shots. The results indicated that there were more correct responses than incorrect ones for shots taken from the free‐throw line, whereas the number of correct and incorrect shots was equal for three‐point attempts. Participants showed different visual strategies depending on the shooting condition. During the three‐point shot, they exhibited more saccades, with greater peak velocity and amplitude compared to the free‐throw condition. Additionally, participants took almost 800 milliseconds less to complete the three‐point shot than the free‐throw, as there were no spatial‐temporal constraints. In conclusion, the high amplitude and peak velocity of the eye movements, along with the reduced time spent preparing for the shot, may have led to more errors in three‐point shots compared to free throws. These findings suggest the need to identify and develop training exercises that effectively refine a shooter's control over release velocity. One potential approach may involve emphasizing feedback from visual cues that are more directly related to release velocity rather than focusing the shooter's attention on specific body movements.

## Highlights

- Athletes tend to provide more correct responses when making shots from short distances than from long distances.
- They also take nearly 800 milliseconds less time to perform long shots than short ones.
- Additionally, as the distance from the basket increases, the number of saccades with greater amplitude and faster peak velocity also increases.

## 1 Introduction

In basketball, shooting from the free throw line, which is positioned 4.60 m away from the basket, appears to be easier than making a shot from the three‐point arc, where the distance to the hoop is 7.24 m. This 3‐m difference significantly impacts shooting success rates: the best free‐throw shooters achieve an impressive 93% success rate, whereas the top three‐point shooters only reach 44% (nba.com/stats [https://www.nba.com/stats/leaders?StatCategory=FG3_PCT&PerMode=Totals]).

Previous researchers have attempted to understand the discrepancy in success between shooting distances by investigating and comparing performance under various conditions, including the release angle and velocity of the ball, proximity to one or more opponents, shooting technique, jumping strategies, and the visual strategy employed by the athletes. Miller and Bartlett (1996) found that the angle and velocity of the ball are conditioned by the distance from the basket, highlighting that these factors determine the outcome of the shot. Breslin et al. (2010) demonstrated that the shot's accuracy decreases as the distance from the basket increases. Slegers et al. (2021) found that as distance and shooting technique changed from free‐throw to three‐point shots, the necessary ball release velocity increased by 24% and lower limb movement and velocity significantly increased. Podmenik et al. (2017) and Okazaki and Rodacki (2012) have also shown that as distance increases, joint angular velocities increase significantly. Combined, these results demonstrate that as the shooting distance increases, both release and limb velocities also increase. Moreover, the higher release velocity impacts the velocity at which the ball touches the hoop, increasing the probability of making an imperfect shot.

The temporal sequence to follow to make an efficient and efficacious skill is to know the precise moment the ball has to be released, at what angle and speed, and where the player has to look during the shooting sequence (Hamilton and Reinschmidt 1997). Amaro et al. (2023), comparing the effect of basketball shooting from different positions and distances, found that when athletes were closer to the basket, in the case of the free throw, the fixation time values and the number of fixations were greater compared to three‐point shots. Marques et al. (2023) found an equal fixation duration in professional basketball players when throwing the ball from different distances (6.80 vs. 4.23 m), and even if it was not analyzed, the number of fixations was higher when the shots were done close to the basket. In their meta‐analysis, Zhao et al. (2024), exploring the relationship between eye movement parameters and free throw scoring, concluded that, within a specific time frame, an increased number of fixations negatively impacts the success rate, whereas longer fixation durations are related to higher scoring percentages. Considering the articles published so far, it appears that the effectiveness of various visual strategies in increasing success rates as the distance increases remains unclear.

In team sports, such as soccer, handball, basketball, volleyball, and others, visual skills have a direct influence on the outcome of the match. More precisely, what has been observed in accuracy techniques is the quality of the visual strategy employed. The visual system influences athletes' posture and performance, influencing their vision on the accuracy of the shot (de Oliveira et al. 2007). Probably, the longer distance involved in a three‐point shot could increase peripheral visual noise and reduce accuracy compared to a free throw in which the basket covers a significant portion of the athlete's foveal receptive field. The influence of visual external perturbations, such as limited time, noise, and the movements of teammates and opponents, can affect the athletes' goal‐oriented focus, thereby influencing their performance (Kostrna 2022). Throwing a ball into a hoop with a diameter of 46 cm (18 inches) is a precision aiming task in which the optimal visual strategy, such as the quiet eye (QE), could reveal intraindividual (e.g., successful vs. unsuccessful tasks) as well as interindividual (e.g., experts vs. novices) differences in motor performance (Vickers 1996). The QE is defined as the last fixation or tracking gaze on a specific cue, made before the critical movement starts. The QE represents a state of optimal attentional control in which the visual focus is maintained at critical cues, thereby blocking both internal and external distracting stimuli (for more information, see Vickers and Lewinski 2012). This, in turn, allows sufficient cognitive resources to be allocated to movement parameterization processes, resulting in superior motor performance (Piras and Vickers 2011). Considering the definition, the QE refers to the last fixation before the movement starts. Until now, most of the studies have defined the QE period relative to a single critical or final motor phase. The same Joan Vickers, who published the first paper on QE (Vickers 1996), has intended that all QE periods should be considered within a motor task. She also showed that during the three‐point shot, it is very hard to reduce the eye movement amplitude until the ball is released (Vickers et al. 2019). For this reason, it is necessary to identify all the visual strategies that contribute to the shooting action.

In the last decade, the interest in microsaccades and other small saccades during fixation has increased, especially their role during action‐perception tasks in sports contexts (Piras and Raffi 2023). It seems that microsaccades may serve varied functions during fixation, just as saccades do during exploration. Microsaccades are useful during the finer examination of the foveated stimulus (Poletti 2023). Saccades are used to explore the scene larger than 2° of the visual angle, shifting the fovea on potentially interesting and relevant stimuli. In natural settings, it seems that microsaccades are not involuntary, uncontrolled movements but rather voluntary, memory‐guided, spatially accurate, and finely controlled (Willeke et al. 2019). Piras et al. (2021b) have investigated the role of saccades and microsaccades in soccer penalties kicked at different distances. Authors found that when the ball was kicked from a greater distance (11m), athletes employed a visual strategy with more saccades compared to penalties kicked from a shorter distance (6m), which showed longer fixations and higher microsaccade rates.

During close‐range shots, the athlete has no external distractors, thereby avoiding a shift in focus of attention to other targets. Even considering the character of the free throw, which is an unopposed task during the game, athletes must remain concentrated and focused on the target to achieve good accuracy over a significant time interval (Piras et al. 2024). Athletes are completely focused on the basket. allowing for a greater concentration, and thus, the fixation durations can be higher. Following this reasoning, if athletes make a three‐point shot as a free throw, without an opponent, we can evaluate the consequence of distance in influencing the visual strategy and the outcome of the shots. The distance influences the dimension of the images perceived; closer objects require both foveal and parafoveal stimulation to capture information, whereas distant objects are perceived only through foveal vision, where saccade amplitudes may scale with scene size (Piras et al. 2021a). Moreover, it can be hypothesized that the variations in accuracy among elite athletes across different shooting distances may be linked to challenges in maintaining stable vision while targeting a distant object. Consequently, athletes might employ distinct visual strategies, utilizing larger saccades for longer shooting distances compared to shorter ones due to the reduced perceptual size of the basket. Following this reasoning, we hypothesize that an increased distance between the shooter and the basket will result in a reliance on information extracted via the fovea, accompanied by a greater number of saccades. Conversely, when the basket is closer, the shooter will utilize peripheral vision to catch external cues, with an oculomotor strategy that relies on fewer saccades. Considering the interplay between eye movements, action‐perception coupling, and attentional focus, the current research investigated the role of eye movements when the ball is released from various distances.

## 2 Methods

### 2.1 Participants

Fourteen (*n* = 14) male basketball players, with a mean age of 21.64 (SD = 3.5), volunteered to participate. Participants were engaged in both free throw and three‐point shots. Participants were 188.21 cm in height (SD = 9.7) and 83.86 in body weight (SD = 12.3). They played basketball for 15 years, with a mean of 5.6 training sessions and 11 h of training per week. All participants had normal or corrected‐to‐normal vision. After receiving both oral and written information concerning the study protocol, all participants signed an informed consent form to participate in the study. The study was approved by the Bioethics Committee of our University.

### 2.2 Apparatus

The EyeLink II (SR Research), a video‐based eye‐tracking system, was used to record eye movements binocularly. The device comprises two miniature cameras mounted on a leather‐padded headband. Pupils were tracked at 500 samples/s with a gaze resolution of < 0.005° and noise limited to < 0.01°. Calibration and validation of the eye tracker were performed at the beginning of the experiment and after every 10 throws. Drift correction was applied to correct the offset of the raw eye position data after every shot. Calibration and validation of the system were repeated every time a possible measurement error occurred due to participant movement. The accuracy of eye position was checked after every throw, and if necessary, a drift correction was performed. Practice, calibration, validation, and data collection took ∼20 min per participant.

One inertial sensor (Cometa Systems, Italy) was placed on the dorsal face of the right hand to collect the exact time participants made the throw. The sensor was synchronized with the EyeLink system to have corresponding eye and hand movement data.

### 2.3 Procedure

Participants visited our laboratory twice, following a randomization order, to perform one day the free‐throw shots and another day the three‐point shots in a counterbalanced order. The goal of counterbalancing is to ensure internal validity by controlling for potential confounds arising from sequence and order effects.

After a warm‐up, a pretest was performed without the eye tracker, followed by the fitting of the eye tracker and taking 3–5 practice trials until the participant became comfortable. Participants also wore a plastic swimming cap to increase friction between the helmet and the head and thus reduce the helmet's movement relative to the head.

#### 2.3.1 Free‐Throw Shots (FT)

Participants stand behind the free‐throw line, located 4.60 m from the basket, and shoot a ball, ∼24 cm in diameter and ∼610 g in weight, into a hoop of ∼46 cm in diameter. The hoop is located directly in front, at a height of 3.05 m from the floor. In front of a basketball hoop, wearing the eye tracker and the inertial sensor, participants made 20 free throws interspersed with 10 min of rest after every 10 shots.

#### 2.3.2 Three‐Point Shots (3P)

In the same court, using the same ball, and with the same basketball hoop in front of them, participants wore the eye tracker and the inertial sensor. They then made 20 three‐point shots, interspersed with 10 min of rest after every 10 shots. The three‐point shots were taken from behind the three‐point arc, a semicircle located 6.75 m from the hoop (FIBA rules).

### 2.4 Statistical Analysis

The shooting sequence was defined as the time interval from the trial start, identified with the inertial sensor on the participant's hand, to the frame in which the ball reached the hoop, identified with the EyeLink scene camera positioned above the participant's head.

Repeated‐measures ANOVA was used to analyze subject‐level shooting accuracy, expressed as the number and percentage of correct and incorrect shots within each shooting condition. The duration of the shooting sequence was analyzed with repeated measure ANOVA in which condition (FT; 3P) and response accuracy (correct; incorrect) were the within‐subjects factors.

We defined microsaccades as small eye movements with a visual angle of less than 1° and with the same peak velocity‐to‐amplitude curve as large saccades. We applied the Engbert–Kliegl algorithm (2003) to identify saccades and microsaccades. To reduce potential noise, we considered only binocular saccades and microsaccades lasting at least three data samples (6 ms), with a velocity threshold detection set at 6. This combination of methods, the EyeLink II system in combination with the Engbert–Kliegl algorithm, appears to be the most commonly used technique to study microsaccades in recent research (Martinez‐Conde et al. 2009).

The rates, amplitudes, durations, and peak velocities of saccades and microsaccades were calculated for each participant during each shot. Repeated measures ANOVA was performed separately to analyze saccade and microsaccade rate, amplitude, duration, and peak velocity. Condition (FT; 3P) and response accuracy (correct; incorrect) were the within‐subjects factor.

All statistical analyses were performed using SPSS, version 22.0 (IBM Corp., Armonk, NY, USA). Partial eta squared (η^2^_p_) was used for effect size estimation with small (∼0.01), medium (∼0.06), and large (≥ 0.14) effects (Cohen 1988). Statistical significance was set at *p* < 0.05. Post hoc testing was corrected using the Bonferroni procedure.

## 3 Results

**FIGURE 1:**
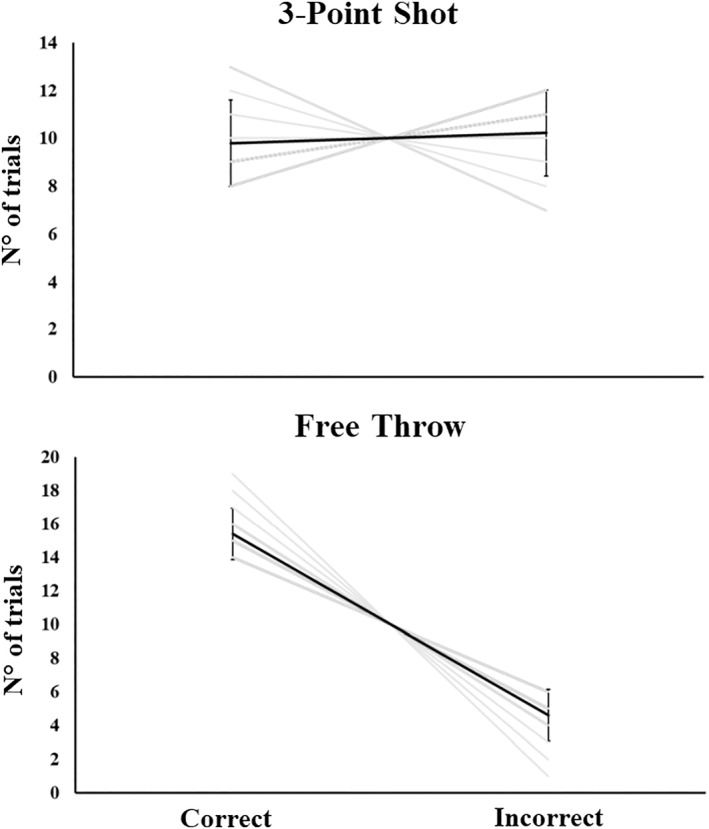
Participants' number of trials (± SD) of both conditions (3‐point shot; free throw) in correct and incorrect responses.

Participants made more correct (mean 15.36 ± 1.6; 77%) than incorrect (mean 4.64 ± 1.6; 23%) free throw shots (*F*_1,13_ = 157.3; *p* < 0.001; η^2^_p_ = 0.92, which indicates a large effect). Meanwhile, we did not find significant differences for the three‐point shots (*p* = 0.66; η^2^_p_ = 0.01, indicating a small effect), where the correct shots were fewer than the incorrect ones (49% vs. 51%, see Figure 1).

The duration of the shooting sequence showed significant differences between conditions (*F*_1, 13_ = 92.1; *p* < 0.001; η^2^_p_ = 0.78, indicating a large effect), with a longer duration during the free throw (mean = 2709 ± 59.8 ms) compared to the 3‐point shot (mean = 1897 ± 59.8 ms). No significant effects were observed considering response accuracy.

**FIGURE 2:**
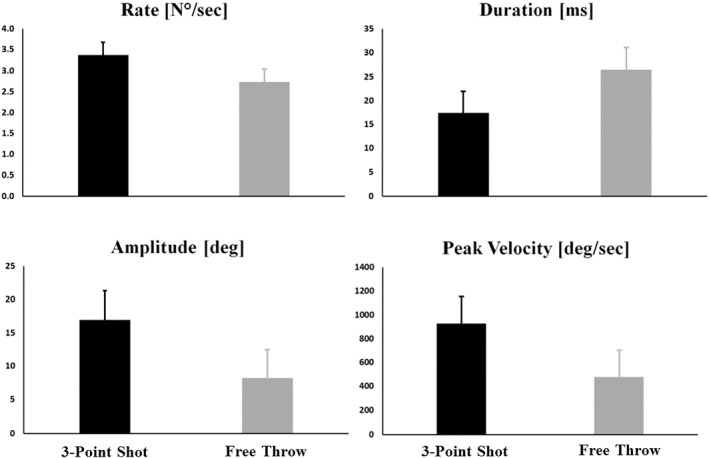
Histograms represent the values (mean ± SD) of saccade characteristics (rate, duration, amplitude, and peak velocity) in both 3‐point shot (black) and free throw (white) conditions.

The algorithm applied to identify saccades and microsaccades reported that, during the three‐point shots, only eye movements greater than 1 degree of visual angle and faster than 100°/sec were identified. For this reason, microsaccades found during the free throw were mixed with saccades and analyzed together. The rate, duration, peak velocity, and amplitude of saccades are shown in Figure 2. Analysis revealed significant differences between conditions for all eye movement parameters examined. Saccades showed significant differences between conditions for rate (*F* _1, 13_ = 4.9; *p* = 0.035; η^2^_p_ = 0.16 indicating a large effect), duration (*F*_1, 13_ = 18.0; *p* < 0.001; η^2^_p_ = 0.40 indicating a large effect), peak velocity (*F*_1, 13_ = 11.4; *p* = 0.002; η^2^_p_ = 0.30 indicating a large effect), and amplitude (*F*_1, 13_ = 20.8; *p* < 0.001; η^2^_p_ = 0.44 indicating a large effect). During the three‐point shot, participants exhibited more saccades of shorter duration, with greater amplitude and peak velocity than shots made from the free‐throw line (see Figure 2). No significant effects were observed considering response accuracy.

**FIGURE 3:**
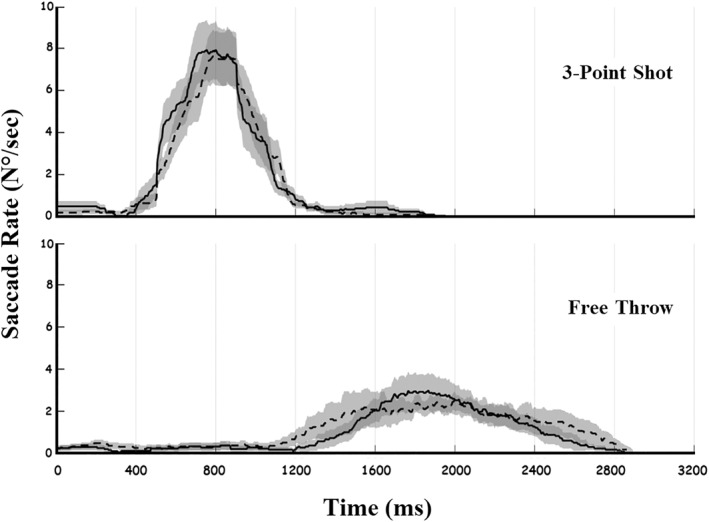
The time course of saccade rates directed to the right (dashed lines) and to the left (solid lines), with the shaded area around each curve representing the standard error of the mean (SEM), in both 3‐point shot (upper panel) and free throw (lower panel) conditions. Rates were computed for each participant using a 200‐ms moving time window and then averaged across all participants.

Additional analysis was done to investigate the temporal sequence of saccades (see Figure 3). During the three‐point shot, saccades reached the highest level, 800 ms, after the trial had started. Eyes were slower during the free‐throw sequence, reaching the saccades' peak 1800 ms after the trial started.

## 4 Discussion

The purpose of this study was to examine the role of eye movements during basketball shooting from various distances. Participants shot 20 times from the three‐point line and another 20 from the free‐throw line. Results showed more correct than incorrect responses during shots taken from the free‐throw position and an equal number of correct and incorrect shots when the ball was thrown from the three‐point position.

Compared to the free throw, which is performed under stable and predictable conditions, the three‐point shot is often executed under different conditions, such as after a dribble, under defensive pressure, and from different positions concerning the hoop. Beyond physical mechanics, the three‐point shot also places significant demands on the athlete's perceptual and cognitive systems (Alemanno et al. 2025). Considering all the other characteristics highlighted above, we intended to exclude all the shooting differences, focusing only on the shooting distance. Therefore, we have asked our participants to take the three‐point shot as they do for the free throw: from a fixed position, without adversarial constraints, where the athlete can focus their visual attention toward the hoop without any spatial‐temporal demands, taking all the time needed to make a successful shot. We acknowledge that this procedure compromises the ecological validity of the three‐point shot, particularly in terms of correct and incorrect results. However, it was the only way to eliminate other factors that influence this type of shot.

In addition to the higher percentage of correct shots in the free throw, we discovered the different visual strategies used by the participants. In the three‐point shot, participants performed more saccades with a greater peak velocity and amplitude than during the free‐throw condition. Furthermore, the surprising result was the time of execution of the three‐point shot. The participants took almost 800 ms less to perform the three‐point shot than the free throw, considering they had no spatial‐temporal constraint. One justification could be that the release speed conditions the shooting distance. The earlier release time gave rise to an earlier rotation of the shoulder axis, which facilitates accuracy by aligning the elbow and wrist joints with the eyes (Miller and Bartlett 1996). The required ball release velocity increased as the shooting distance changed from the free throws to the three‐point shots. Performance is almost exclusively determined by the shooters' ability to control their release velocity through attempts. Since, under a wide range of conditions, players' performance may be impaired by their inability to compensate for variability using elbow‐wrist joint proprioception (Slegers et al. 2021). Perhaps the high amplitude and peak velocity of the eye movements, combined with the short time spent preparing the shot, caused our participants to miss significantly more three‐point shots than free throws. Research has demonstrated that athletes' eye movements during the final phase of shooting involve the rapid integration of spatial and motion‐related information, such as the distance and angle of the basket and their body positioning to calibrate their motor output (Han et al. 2025). Gaze stability (e.g., maintaining prolonged focus on the rim during throws) provides athletes with additional time to process critical visual information and optimize movement planning. This mechanism enables athletes to refine their movement execution through enhanced cognitive readiness and the suppression of distractions. These results align with those found by Piras et al. in basketball free throws (2024) and Piras in three‐point shots (2024). The authors found that during free throws, their participants (near‐experts and amateurs) needed 2000 ms to make the shot (without considering the ball flight). During the three‐point shots, avoiding the opponent's intervention, participants needed 1600 ms to make the shot (without considering the catching phase). Supporting this conclusion is the vast literature on the role of the QE in elite sports. The QE approach has been studied in several sports (for more information, see Vickers 2016), and the original findings have been replicated many times as meta‐analyzed by Lebeau et al. (2016). The QE technique is characterized by a continuous gaze on a target before initiating the critical movement. It is crucial in improving accuracy in various sports and tasks by focusing attention and enhancing visuomotor integration. It helps athletes stay calm under pressure, gather information, and execute movements precisely. Training programs that effectively increase the QE period enhance the gaze strategy, thereby improving performance (Harle and Vickers 2001). Special consideration should be given to the study by Klostermann et al. (2018), in which, during an undefended shot taken from the free throw line, highly skilled players exhibited a similar QE duration for both hit and miss shots. This result might simply be a consequence of the situation's or the motor task's specificity, in line with the current discussion on task‐specific changes in gaze behavior (Dicks et al. 2010). Results related to perception‐action coupling are tied to the actual demands of the motor task, which may not always be replicable in experimental settings.

During QE, our eyes fixate at a visual angle of 1–3°. It has been found that different types of eye movements fit within this visual window (e.g., microsaccades). These small micromovements may be a favorable strategy for a given task. Piras et al. (2021a), during penalties kicked at different distances, found that expert goalkeepers, in the long distance (11 m), used a visual strategy with more fixations, and consequently greater saccade rates in comparison to penalties made from short distance (6 m), where they exhibited fewer fixations and higher microsaccade rates. Authors suggest that this could be due to the player's and the ball's different distances, which influence the dimension of the images perceived; as long as the target distance is considerable, the gaze can be frequently shifted between the cues because the costs of saccades are low. On the other hand, closer targets require both foveal and parafoveal stimulation to capture information, as the costs of saccades are high. Saccades are reduced, and to acquire visual information, it is necessary to monitor different cues also with the parafovea (likely using microsaccades or small saccades). This coordination of eye and hand movements has several consequences. Directing eye movements toward an object and foveating on it (e.g., with microsaccades), the eyes provide spatial information for the hands (L. Zhang et al. 2022). Generally, it is more accurate when the gaze remains fixed on the intended target, thus avoiding the extra processing needed for spatial updating during gaze shifts while pointing (Crawford et al. 2004). Therefore, it is conceivable to expect that if inadvertent saccade intrusions disrupt this coupling, subsequent motor actions would suffer delays and a lack of coordination (Terao et al. 2017). The relationship between saccade frequency and motor performance is complex, with potential benefits and disadvantages depending on the context. So, it is reasonable to think that an excessive number of saccades, especially those of greater amplitude and not directed toward the target point, can negatively impact accuracy. On the contrary, higher small saccade rates allow athletes to maintain an extended duration of programming (goal‐directed control), focusing attention on the target and minimizing distraction from other environmental cues (stimulus‐driven control) (Eysenck et al. 2007; Wilson et al. 2009). The spatiotemporal features of saccadic eye movements may reflect an optimum sampling method by which the brain discretely acquires visual information and can discriminate between athletes who use a fixation before the motor action and athletes who move the eyes to catch more visual cues before to make the critical movement (Piras et al. 2021a, 2021b; Timmis et al. 2018; Z. Zhang et al. 2022).

In conclusion, the results of the present study suggest that the distance from the target modulates saccade characteristics, which, in turn, may influence the outcome of the action. When the distance to the target was greater, participants made more saccades of greater amplitude and peak velocity than in conditions with a closer target. Moreover, during the aiming task, saccades of lower amplitude and peak velocity were related to higher accuracy. It appears that these subtle eye movements may enhance action perception, aiding athletes during the critical moment preceding the motor response and directing attention toward the key cues related to the perception of the motor outcome. These results highlighted the need to identify and develop training exercises that demonstrate efficacy in refining a shooter's release velocity control. One potential approach to achieving good results may be to place greater emphasis on training feedback from visual cues that are more directly related to release velocity, rather than focusing on specific body motions. Moreover, it is essential that athletes stabilize their gaze on the external target, reducing the number of saccades, in order to focus their attention on the force to apply to the ball. This could reduce the potential interference between saccade eye movements and hand movements (Richardson et al. 2013).

## Conflicts of Interest

The author declares no conflicts of interest.

## Data Availability

Research data are not shared.
